# *Penicillium marneffei*-Stimulated Dendritic Cells Enhance HIV-1 *Trans*-Infection and Promote Viral Infection by Activating Primary CD4^+^ T Cells

**DOI:** 10.1371/journal.pone.0027609

**Published:** 2011-11-16

**Authors:** Yan Qin, Yuye Li, Wan Liu, Renrong Tian, Qianqian Guo, Shaoyou Li, Hongbin Li, Daojun Zhang, Yongtang Zheng, Li Wu, Ke Lan, Jianhua Wang

**Affiliations:** 1 Key Laboratory of Molecular Virology and Immunology, Institute Pasteur of Shanghai, Chinese Academy of Sciences, Shanghai, China; 2 The First Affiliated Hospital of Kunming Medical College, Kunming, China; 3 Key Laboratory of Animal Models and Human Disease Mechanisms of Chinese Academy of Sciences and Yunnan Province, Kunming Institute of Zoology, Chinese Academy of Sciences, Kunming, China; 4 Department of Veterinary Bioscience, Center for Retrovirus Research, Ohio State University, Columbus, Ohio, United States of America; Jiangsu University, China

## Abstract

*Penicillium marneffei* (*P. marneffei*) is considered an indicator pathogen of AIDS, and the endemicity and clinical features of *P. marneffei* have been described. While, how the co-infection of *P. marneffei* exacerbate deterioration of the immune response remains poorly understood. Here we isolated *P. marneffei* from the cutaneous lesions of AIDS patients and analyzed its effects on HIV-1-dendritic cells (DCs) interaction. We demonstrated that the monocyte-derived dendritic cells (MDDCs) could be activated by both thermally dimorphic forms of *P. marneffei* for significantly promoting HIV-1 *trans*-infection of CD4^+^ T cells, while these activated MDDCs were refractory to HIV-1 infection. Mechanistically, *P. marneffei*-activated MDDCs endocytosed large amounts of HIV-1 and sequestrated the internalized viruses into tetrapasnin CD81^+^ compartments potentially for proteolysis escaping. The activated MDDCs increased expression of intercellular adhesion molecule 1 and facilitated the formation of DC-T-cell conjunctions, where much more viruses were recruited. Moreover, we found that *P. marneffei*-stimulated MDDCs efficiently activated resting CD4^+^ T cells and induced more susceptible targets for viral infection. Our findings demonstrate that DC function and its interaction with HIV-1 have been modulated by opportunistic pathogens such as *P. marneffei* for viral dissemination and infection amplification, highlighting the importance of understanding DC-HIV-1 interaction for viral immunopathogenesis elucidation.

## Introduction

*P. marneffei* is considered an indicator pathogen for AIDS [1], [2]. It mainly exits endemically in area of South East Asia that causes fever, lymphadenopathy, hepatosplenomegaly and cutaneous lesions [3], [4]. *P. marneffei* has the unique feature among the species of *Penicillium* of being thermally dimorphic for diagnosis, it grows as a mycelium at 25°C, and a soluble red pigment is produced, whereas, at 37°C, it grows as a yeast form [5]. The clinical features of *P. marneffei* in AIDS patients have been well described [6], [7], and like other opportunistic pathogens, the infection of *P. marneffei* would exacerbate deterioration of the immune response and accelerate AIDS disease progression, while the mechanism remains elusive.

Dendritic cells (DCs) play pivotal roles in host defense by initiating innate immunity and bridging adaptive immunity [8], [9], [10]. DCs are widely distributed in the sub-mucosa, yet have been believed to be involved in HIV-1 infection and transmission [9], [11], [12], [13], [14], [15], [16]. The migration property of DCs has been hijacked by HIV-1 for viral dissemination to CD4^+^ T cells by a process that is known as *trans*-infection [8], [17], [18], [19], [20], [21], [22], [23], [24], [25]. The formation of DC-T-cell conjunction, or so-called virological synapses, at which numerous intact viral particles and viral receptors can be recruited, appears to be required for efficient viral transfer [26], [27]. Upon activation by stimuli, such as the bacterial product LPS (lipopolysaccharide), DCs could uptake much more viruses and recruit significantly amounts of viral particles on the virological synapses for enhancement of HIV-1 *trans*-infection [27]. DCs express HIV-1 receptors and can serve as targets for productive HIV-1 replication. Persistent infection of HIV-1 may generate the potential long-lived viral reservoirs in DCs [15], [28], [29]. DCs appear to take vital roles in HIV-1 infection and viral pathogenesis, and a better understanding of the interactions between HIV-1 and DCs would facilitate the elucidation of AIDS pathogenesis.

We hence isolated *P. marneffei* from the cutaneous lesions of AIDS patients and analyzed its effects on HIV-1-dendritic cells interaction. We found that MDDCs could be activated by both dimorphic forms of *P. marneffei* for significantly promoting HIV-1 *trans*-infection of CD4^+^ T cells, and the *Candida albicans* (*C. albicans*), which has been proved to possess the similar capacity [30], was used as control. Increased expression of intercellular adhesion molecule 1 (ICAM-1) was observed on fungus-activated MDDCs, and the tighter DC-T-cell conjunction recruited significant amounts of virus particles for viral transfer. We also found that *P. marneffei*-activated MDDCs efficiently activated resting CD4^+^ T cells through cell-cell contact and thereby could result in more susceptible targets for viral infection. Our findings demonstrate that DC function and its interaction with HIV-1 have been modulated by opportunistic pathogens such as *P. marneffei* for viral dissemination and infection amplification, highlighting the importance of understanding DC-HIV-1 interaction for viral immunopathogenesis elucidation.

## Results

### P. marneffei stimulation promotes the activation of MDDCs

**Figure 1:**
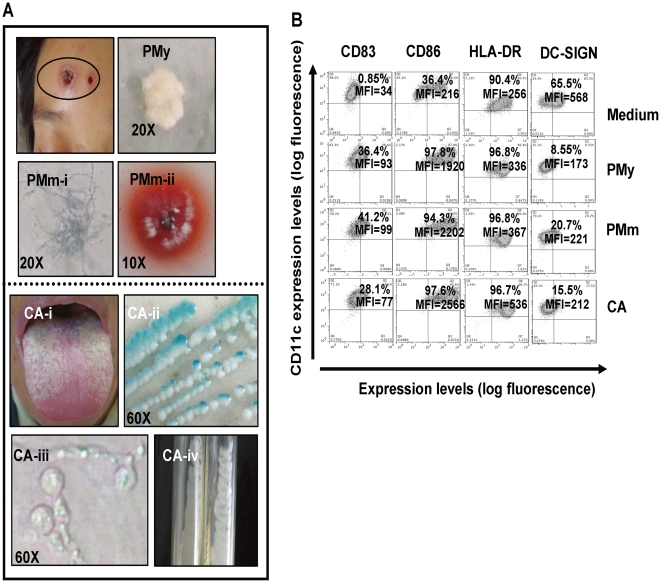
Fungal stimulation induces MDDCs activation. (A) *Ex vivo* culture and characterizations of fungi species associated with HIV-1 infection. *P. Marneffei* was isolated and identified from a skin lesion from an AIDS patient. Sub-cultured *P. Marneffei* showed dimorphisms, with a yeast form at 37°C growth (PMy) and a mycelial form at 25°C (PMm-i); the production of red pigments at 25°C is indicated (PMm-ii). *C. Albicans* was isolated from the tongue of an AIDS patient (CA-i), and *identified with sub-inoculation* in CHROMagar Candida displaying the green color (CA-ii), and in Corn Tween agar showing the formation of sporulation (CA-iii), *C. albicans* was sub-cultured *ex vivo* (CA-iv). (B) Activation of MDDCs was monitored by flow cytometry. Immature MDDCs were treated with heat-killed *C. albicans*, PMy and PMm at a ratio of 1∶10 for 48 h, and medium treatment was used as a control. MDDCs were gated as a CD11c^+^ population, and fungus-stimulated MDDCs showed increased expression of HLA-DR and the co-stimulatory molecules CD83 and CD86, with decreased expression of DC-SIGN, compared with the medium-treated controls. The positive percentages and values of MFI from one representative out of six experiments are indicated.

In current study, the *C. albican* which has been described previously for induce DC activation was used as a control [30]. *P. marneffe* and *C. albicans* were isolated separately from the skin lesions or the tongues of AIDS patients and cultured in Sabouraud agar plates. *P. marneffei* has the unique feature among the species of *Penicillium* of being thermally dimorphic, it grows as a mycelium at 25°C, similar to *Aspergillus* spp, and a soluble red pigment is produced (Figure 1A, PMm-i and-ii), whereas, at 37°C, it grows as a yeast form (Figure 1A, PMy). *C. albicans* was identified with sub-inoculation in CHROMagar Candida (Figure 1A, CA-ii), in Corn Tween agar (Figure 1A, CA-iii) and with the API 20C AUX yeast identification system. These fungi were sub-cultured for amplification and harvested for heat inactivation.

To investigate the potential activation of DCs by fungi, MDDCs were incubated separately with heat-inactivated *C. albicans*, PMy and PMm at a ratio of 1∶10 of DCs to fungi. The phenotypes of MDDCs were examined by immunostaining of cell surface markers; MDDCs showed high level of CD11c expression and were measured for CD83, CD86 and HLA-DR expression levels. Fungal stimulation significantly increased CD83, CD86 and HLA-DR expression compared with medium-treated cells, indicating of fungus-induced MDDCs activation (Figure 1B), whereas surface expression of a C-type lectin, DC-SIGN (DC-specific intercellular adhesion molecule 3-grabbing nonintegrin), was decreased in fungus-treated MDDCs (Figure 1B). These data suggest that stimulation of *P. marneffei* promotes DCs activation.

### HIV-1 infection of P. marneffei-activated MDDCs is blocked

**Figure 2:**
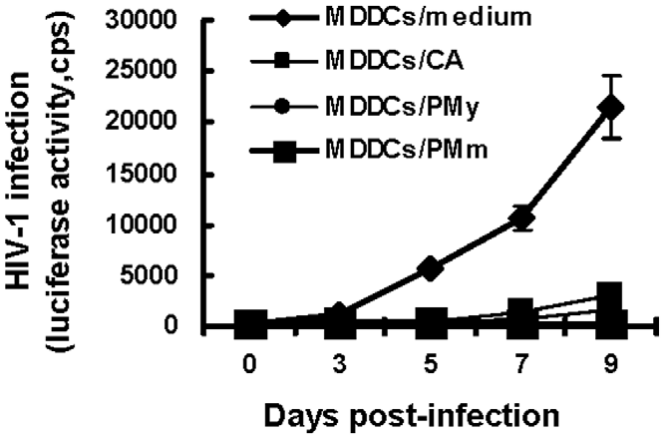
HIV-1 infection is blocked in MDDCs after fungal stimulation. Immature MDDCs were treated separately with heat-killed fungus species *C. albicans*, PMy and PMm or control medium for 48 h, and then were incubated with single-cycle luciferase reporter virus HIV-Luc/JRFL (5 ng of p24^gag^) for 2 h. After washing, HIV-1-pulsed MDDCs were incubated and harvested at the indicated times, and HIV-1 infection was detected by measuring the luciferase activity in cell lysates. Results of one representative experiment out of three are shown. All data are mean ± standard deviation (SD).cps, counts per second.

To examine the effects of the stimulation of *P. marneffei* on HIV-1 infection of MDDCs, fungus-treated MDDCs were inoculated with single-cycle, luciferase reporter HIV-Luc/JRFL (CCR5-tropic), and HIV-1 infection was measured by detecting the luciferase activity in cell lysates at 3-9 days post-infection. HIV-1 infection was fully blocked in all fungus-treated MDDCs compared with medium-treated controls (Figure 2), and the detected luciferase activities decreased by at least 75% at 5, 7 and 9 days post-infection. The *C. albicans*, which has been shown to inhibit HIV-1 replication in DCs, was used as a control [30]. These data suggest that HIV-1 infection in MDDCs is blocked after the stimulation of *P. marneffei*.

### P. marneffei stimulation promotes DC-mediated HIV-1 transmission to CD4^+^ T cells

**Figure 3:**
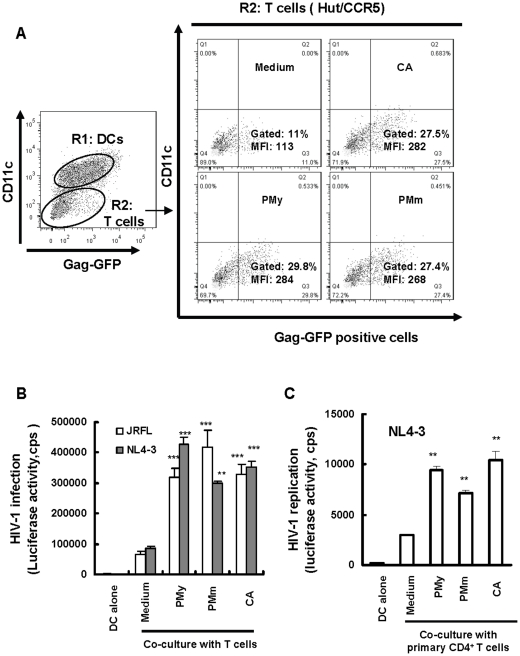
Fungal stimulation promotes MDDC-mediated HIV-1 transmission to CD4^+^ T cells. Immature MDDCs were treated with heat-killed fungi species as described in Figure 2. After pulsing with 40 ng p24^gag^ amounts of HIV-1 VLPs (A), or with 5 ng p24^gag^ amounts of pseudotyped HIV-Luc/NL4-3 or HIV-Luc/JRFL(B and C), CD4^+^ T target cells were added for co-culture, HIV-1 VLP transfer was detected either by flow cytometry after 30 min (A), or by measuring HIV-1 infection after 3 days co-culture (B and C). (A) Enhanced viral association with Hut/CCR5 cells mediated by fungus-stimulated MDDCs. Hut/CCR5 cells (CD11c^-^) were gated from co-culture, and viral association was measured by detecting Gag-GFP level by flow cytometry. The positive percentages and the values of MFI from one representative out of three experiments are shown; (B, C) Increased HIV-1 *trans* infection mediated by fungus-stimulated MDDCs, either with Hut/CCR5 cells (B) or PHA-activated autologous primary CD4^+^ T cells as target cells (C). Asterisks indicate significantly enhanced HIV-1 *trans* infection mediated by fungus-treated MDDCs compared with that of medium-treated cells (**P <0.01, ***P <0.001, paired *t* test); Results of one representative experiment out of four are shown. All data are means ± SD. cps, counts per second.

To determine the capacity for DC-mediated HIV-1 transmission after *P. marneffei* stimulation, MDDCs were treated with heat-inactivated fungi as above and GFP-containing HIV-1 VLPs were used to measure viral transmission efficiency using flow cytometry. HIV-1 VLP-loaded MDDCs were co-cultured with human CD4^+^ T-cell line Hut/CCR5 for 30 min, Hut/CCR5 cells with the CD11c^-^ staining were gated, and Gag-GFP-associated cells were quantified. The level of GFP association on Hut/CCR5 cells increased from 11% in medium-treated controls to 27–30% in fungus-activated MDDCs, the MFI values showed an over 2-fold increase (Figure 3A). Thus, the fungus, including *P. marneffei* and the *C. albicans* control, can promote MDDC-mediated HIV-1 transfer to CD4^+^ T cells.

Viral *trans* infection was also quantified by using the DC-T-cell co-culture system as described previously [27], [31], [32]. Pseudotyped single-cycle, luciferase reporter HIV-1 was used. Hut/CCR5 and activated autologous primary CD4 ^+^ T cells were used as the target cells. Differently treated MDDCs loaded HIV-luc/JRFL or HIV-luc/NL4-3 were co-cultured with target cells for 3 days, and HIV-1 infection was monitored by measuring luciferase activity. Fungus-stimulated MDDCs significantly enhanced the capacity to mediate HIV-luc/JRFL or HIV-luc/NL4-3 *trans* infection of HutCCR5 cells, there was a 4.8- to 6.5-fold increase in luciferase activity (Figure 3B), when activated primary CD4^+^ T cells were used as target, fungus-stimulated MDDCs enhanced HIV-luc/NL4-3 *trans-*infection of primary CD4^+^ T cells approximately 2-3- fold (Figure 3C). *C. albicans*, having been shown previously to promote DC-mediated HIV-1 transmission, was used as a positive control [30]. Together, these results indicate that MDDCs stimulated by PMm and PMy enhanced their capacity to mediate HIV-1 *trans* infection of CD4^+^ T cells.

### Enhanced endocytosis and altered intracellular trafficking of HIV-1 in fungus-activated MDDCs

**Figure 4:**
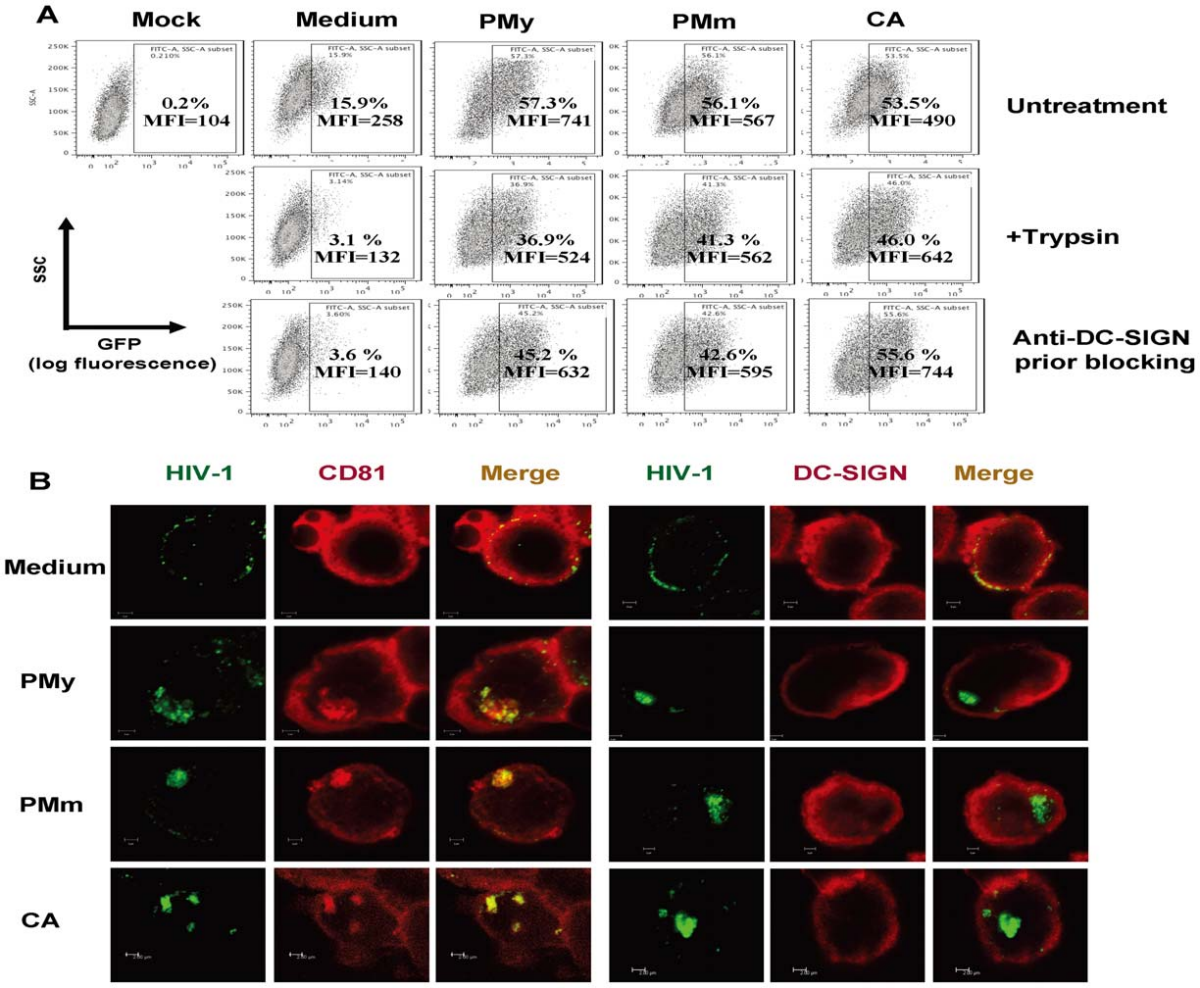
Fungus-treated MDDCs enhance HIV-1 endocytosis and alter viral intracellular sequestration. (A) Enhanced HIV-1 endocytosis in Fungus-treated MDDCs. Immature MDDCs were treated with heat-killed fungi as described above. HIV-1 VLPs (40 ng amounts of p24^gag^) were added for 2 h incubation, and some samples were prior-blocked with anti-DC-SIGN antibodies, and trypsin treatment for 5 min at room temperature was used to remove surface-bound virus. Gag-GFP level was detected by flow cytometry, and the positive percentages and the calculated MFI values from one representative out of four experiments are denoted. (B) Internalized HIV-1 VLPs are sequestrated in CD81^+^ DC-SIGN^-^ compartments in fungus-stimulated MDDCs. Immature MDDCs were treated with heat-killed fungi and pulsed with HIV-1 VLPs as described for panel A, MDDCs were fixed and immunostained for DC-SIGN or CD81, and cells were observed by confocal microscopy. Scale bars, 2 µm.

LPS-activated DCs potently enhance HIV-1 *trans* infection and the endocytosis of large amounts of viruses, and the harboring of intact viruses in non-classical multiple vesicular bodies might provide viruses with a means to escape from intracellular proteolysis [27], [33], [34]. To investigate whether fungal stimulation similarly affect on viral endocytosis, fungus-stimulated MDDCs were pulsed with HIV-1 VLPs for 2 h. Trypsin was used to strip cell-surface-bound virus particles, and the internalized viral particles were quantified by detection of Gag-GFP by flow cytometry. Numerous viruses were internalized by fungus-stimulated MDDCs (Figure 4A). Gag-GFP was demonstrated in 53.5–57.3% of MDDCs stimulated by *C. albicans*, PMy or PMm, compared with 15.9% in medium-treated immature MDDCs, and the calculated MFI of GFP also exhibited a two-fold increase relative to that in medium-treated controls. The majority of MDDCs-associated viruses could not be removed by trypsin digestion. Blocking with anti-DC-SIGN antibodies before VLP pulsing did not inhibit viral uptake, suggesting a DC-SIGN-independent endocytosis process (Figure 4A). By contrast, as shown previously [27], immature MDDCs bind HIV-1 mainly through surface-expressed DC-SIGN, which can be easily removed by trypsin treatment (Figure 4A).

To better understand intracellular trafficking in fungus-stimulated DCs, HIV-1 VLP-pulsed MDDCs were visualized by confocal microscopy with immunostaining (Figure 4B). Fewer viruses were evenly distributed on the surface or were internalized in medium-treated immature MDDCs, whereas many viral particles were endocytosed and concentrated into the CD81^+^ DC-SIGN^-^ compartments in fungus-treated MDDCs. These results are consistent with previous observations that LPS-stimulated MDDCs sequester intact HIV-1 in a specialized and tetraspanin CD81^+^ compartments [31], [33], [34], [35], [36]. These data suggest that the stimulation by PMy, PMm or *C. albicans* largely promotes HIV-1 endocytosis and sequestration within the tetraspanin CD81^+^ compartments of fungus-activated DCs.

### Fungus-activated MDDCs increase ICAM-1 expression, and facilitate DC-T cell contact formation and viral concentration in virological synapses

**Figure 5:**
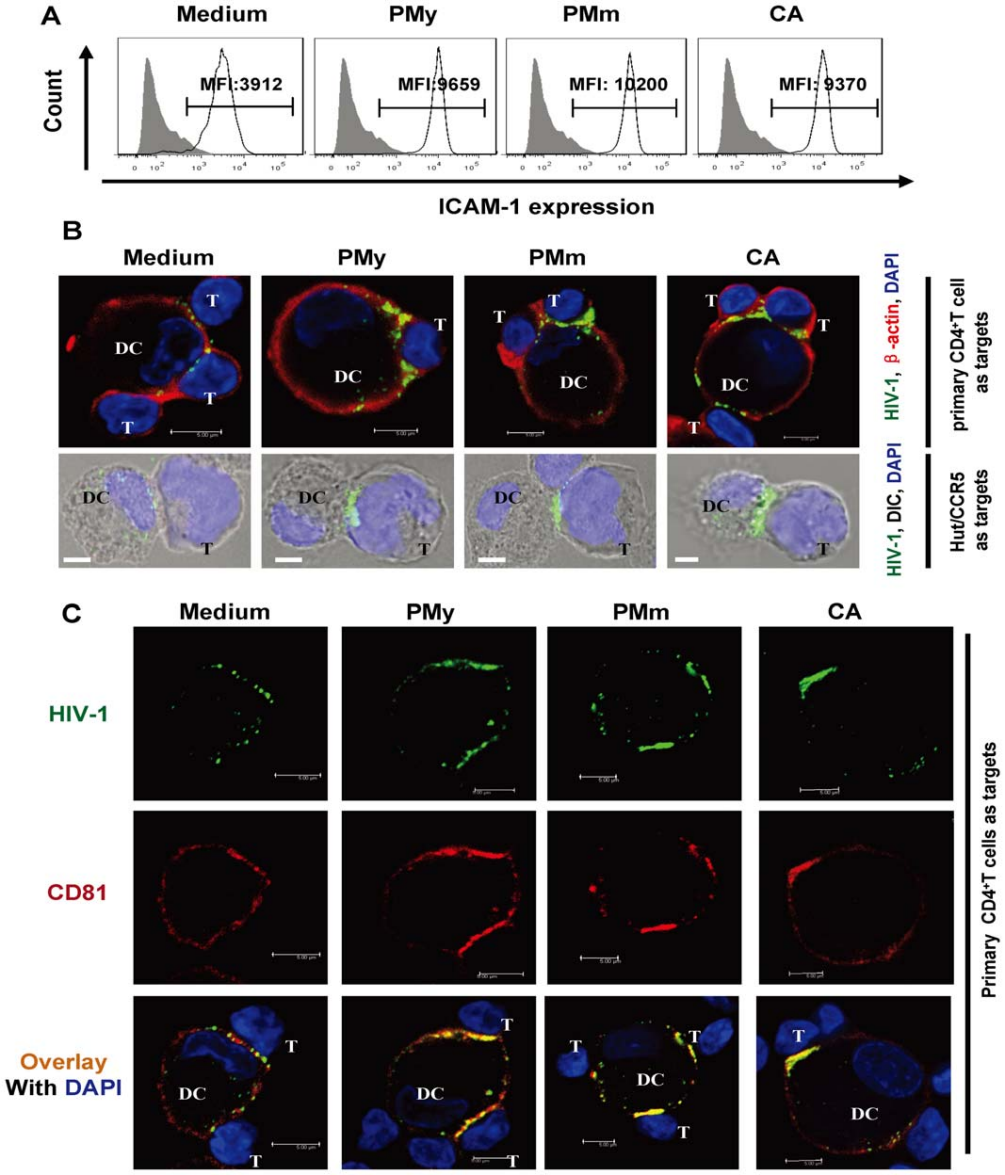
Fungus-stimulated MDDCs increase surface expression of ICAM-1 and facilitate the formation of virological synapses between MDDCs and CD4^+^ T target cells. (A) Increased ICAM-1 expression on fungus-stimulated MDDCs. Immature MDDCs were treated separately with heat-inactivated *C. albicans*, PMy and PMm or medium. The surface level of ICAM-1 was detected by flow cytometry. The MFI values are shown. (B, C) Enhanced viral concentration on the contact sites between MDDCs and T cells. Immature MDDCs were treated with fungi as above, HIV-1 VLPs (40 ng p24^gag^) were added for 2 h incubation, and Hut/CCR5 or PHA-activated autologous primary CD4^+^ T cells were added as target cells for 30 min co-culture. Cells were fixed and observed by confocal microscopy. β-actin (B) or tetraspanin CD81 (C) were stained and shown in red. Nuclei were stained by DAPI. DIC, differential interference contrast. Scale bars, 5 µm.

The virological synapses have been demonstrated to provide the most efficient route for HIV-1 transfers between contacting cells [26], [34], [37]. The ICAM-1-LFA-1 interaction has been proved to be involved in the formation of DC-T-cell conjunction and contribute to efficient HIV-1 transfer [32]. To investigate further the mechanism by which fungal treatment enhances viral transfer, ICAM-1 expression on the cell surface was measured. Stimulation with heat-killed PMy, PMm and *C. albicans* enhanced ICAM-1 expression on MDDCs by 2.4- to 2.6-fold (Figure 5A), which indicates the potential for tighter cell conjunction formation. The formation of virological synapses was visualized by confocal microscopy, and Hut/CCR5 or activated primary CD4^+^ T cells were used as target cells (Figure 5B). Many viral particles were efficiently concentrated at the fungus-stimulated DC-T cell contact sites to form virological synapses. In more detailed analysis of the staining, the tetraspanin molecule of CD81 was recruited to the contact sites (Figure 5C), which suggests a potential role of CD81 in HIV-1 trafficking. Taken together, these data demonstrated that the enhanced ICAM-1 expression and virological synapses account for increased HIV-1 *trans*-infection mediated by fungus-stimulated MDDCs.

### Fungi facilitate DC-induced activation of resting CD4 ^+^ T cells and promote viral infection by providing more permissive cell targets

**Figure 6:**
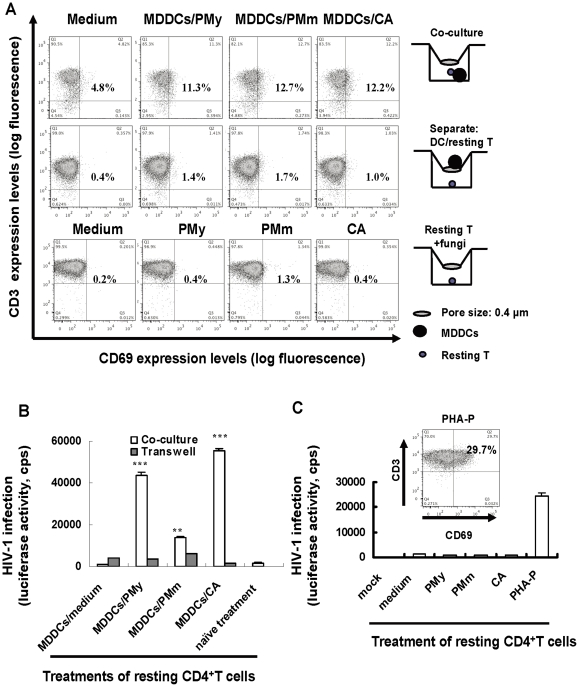
Fungus-loaded MDDCs facilitate the activation of resting CD4^+^ T cells and promote HIV-1 infection by activating more permissive cell targets. MDDCs were pulsed separately with heat-killed *C. albicans*, PMy, PMm or medium control for 2 h, then were co-cultured with same numbers of allogeneic resting CD4^+^ T cells. Transwell plates with membrane pore sizes of 0.4 µm were used to separate MDDCs from T cells. The activation of resting T cells directly stimulated with fungi was also checked. After additional 48 h incubation, T cells were gated by a CD3^+^ cell population to detect CD69 expression by flow cytometry (A). CD4^+^ T cells were purified with magnetic micro-beads, pulsed with 5 ng p24^gag^ amounts of pseudotyped HIV-Luc/NL4-3 for 2 h, and HIV-1 infection was detected 3 days later by measuring the luciferase activity in cell lysates (B, C). Asterisks indicate significantly enhanced HIV-1 replication in T cells activated by fungus-stimulated MDDCs during co-culture, relative to that in medium-treated MDDCs (**P <0.01, ***P <0.001, paired *t* test). Direct treatment of heat-killed fungi neither activated resting T cells nor enhanced T-cell susceptibility to HIV-1 infection. PHA-P was used as a positive control for non-specific activation of resting T cells, and the positive percentages of CD69 are shown in (A) and (C). Results of one representative experiment out of three are shown. Data are mean ± SD.

DCs can efficiently active naïve T cell, and the activated T cells can provide more permissive targets for robust viral infection. To examine the potential effects of fungi on DC-induced CD4^+^ T cell activation and HIV-1 infection of T cells, MDDCs were pulsed with heat-killed fungi or control medium for 2 h. After washing, MDDCs were co-cultured with allogeneic resting CD4^+^ T cells for an additional 48 h. T-cell activation was monitored by detection of CD69 expression in gated CD3^+^ cells. Overall, fungus-pulsed MDDCs facilitated T-cell activation (Figure 6A). In the presence of fungi-pulsed MDDCs, CD69 was expressed on the surface of around 12% of T cells, compared with 4.8% of T cells cultured with MDDCs without fungi (Figure 6A). In order to demonstrate the DC-T cell direct contact is requirement for efficient T activation, the transwell plates with an insert membrane size of 0.4 µm were used to separate the MDDCs from T cells. As expected, much less or non- activation of resting T cells was observed (Figure 6A). Direct stimulation with heat-killed fungi alone induced very little, transient expression of CD69 on resting T cells, or no expression at all, which demonstrated the need for DCs for activation of resting T cells (Figure 6A).

We investigated whether fungus-loaded MDDCs can facilitate T-cell susceptibility to HIV-1 infection. T cells co-cultured with fungi-loaded MDDCs were purified and plused with pseudotyped sing-cycle HIV-Luc/NL4-3 reporter virus, and viral infection was detected 5 days later by measuring luciferase activity in cell lysates. HIV-1 infection was significantly enhanced in primary CD4^+^ T cells activated by fungus-pulsed MDDCs compared with control MDDCs without fungi. Moreover, direct cell-cell contact was required for initiating T-cell susceptibility to viral infection (Figure 6B). Direct treatment of heat-killed fungi did not induce susceptibility of resting T cells to HIV-1 infection. PHA-activated CD4^+^ T cells were used as a positive control, which displayed around 30% cells expressed CD69 expression and supported efficient HIV-1 infection in treated T cells (Figure 6C). These data suggest that fungus-pulsed DCs facilitate the activation of resting T cells and activate more permissive T cells targets for robust HIV-1 replication.

## Discussion

Microbial translocation has been proposed as the cause of systemic immune activation in chronic HIV-1 infection [38]; however, it has not been extensively studied how these co-pathogens speed up deterioration of the immune response. DCs appear to be the common targets for HIV-1 invasion and translocation of other opportunistic pathogens at the mucosa. The functional compromise of DCs by HIV-1 infection is associated with immunosuppression and lack of control of microbial translocation. Given the pivotal roles of DCs in host immunity and viral pathogenesis, the interactions of DCs with HIV-1 have been preferentially targeted for exploiting the potential effects of opportunistic pathogens.

DCs treated with opportunistic pathogens, such as *Malaria hemozoin*, *Mycobacterium tuberculosis*, and *C. albicans*, impairs degradative processing and MHC-II presentation of HIV-1 antigens to CD4^+^ T cells, and alters cytokine secretion, the enhanced DC-mediated viral *trans* infection was also observed during certain opportunistic infections [30], [39], [40], [41], [42]. In those published studies, the synthetic *hemozoin* products, the *M. tuberculosis* cell wall, or the heal-killed *M. tuberculosis* or *C. albicans* laboratory strains was used.

Here, the effects of *P. marneffei* on HIV-1-DC interactions were investigated. The difference is that the used *P. marneffei and the C. albicans* were directly isolated from AIDS patients. Our results demonstrated that both thermally dimorphic forms of *P. marneffei* activated DCs and promoted DC-mediated HIV-1 *trans*-infection of CD4^+^ T cells. Moreover, *P. marneffei* -stimulated DCs could further activate resting CD4^+^ T cells to induce more susceptible targets for HIV-1 infection. Our results have also shed light on the detailed mechanisms for the enhancement of viral spread. We demonstrated that heat-killed *P. marneffei*, along with *C. albicans*, promote viral uptake in MDDCs, altered viral intracellular sequestration, and importantly, facilitated MDDC-T cell contact by increasing ICAM-1 expression and efficiently concentrating HIV-1 particles in virological synapses.

DC activation and altered viral intracellular trafficking are associated with enhanced viral spread [27], [31], [33]. Upregulation of HLA-DR, costimulatory molecules CD83 and CD86, and intercellular molecules on fungus-activated DCs, in general, would encourage DC-T cell conjugate formation. We and other groups have previously reported that increased ICAM-1 expression on DCs correlates with promoted viral transfer, due to stronger DC-T cell interactions through ICAM-1 binding to T-cell-expressed LFA-1 [32], [43]. Fungus-stimulated DCs accelerate viral uptake and sequestrate intact viral particles in non-conventional, non-lysosomal tetraspanin CD81^+^ compartments. The harboring of intact virus into the non-classical multiple vesicular bodies might provide virus a means to escape from the cellular proteolysis [16], [31], [33], [34], [36]. Upon encountering with CD4^+^ T cells, more viruses were recruited on the DC-T cell contacted sites. High levels of endocytosis and altered intracellular trafficking of HIV-1 appear to account for enhanced viral transmission mediated by fungus-activated DCs.

*P. marneffe*-stimulated DCs were less permissive for productive infection, which is consistent with previous reports of LPS and *malaria hemozoin* treatment [40]. However, it remains to be clarified which fungal component(s) is responsible for HIV-1 restriction and the underlying mechanisms. LPS-matured DCs show dis-association of the susceptibility for HIV-1 infection with the capacity for mediating HIV-1 *trans* infection [44]. Post-entry restriction of HIV-1 infection in LPS-induced mature DCs has been noted, and inhibition on the levels of reverse transcription and post-integration have been further identified by using real time PCR quantification of viral DNA and integration [44]. Reduced gene expression, such as for co-receptor CCR5, has been reported to be responsible for impaired productive infection of HIV-1 in *malaria-hemozoin-*treated DCs [40]. Higher levels of APOBEC3G and APOBEC3F (for “apolipoprotein B mRNA-editing enzyme, catalytic polypeptide-like 3G and 3F”) also have been shown to mediate the post-entry block of HIV replication in DCs and LPS can upregulate the expression of APOBEC3G/F [45], [46]. The antiretroviral protein, namely SAMHD1 (SAM domain HD domain-containing protein 1), has been recently identified to inhibit the early step of HIV-1 replication in dendritic- and myeloid cells [47], [48]. It might be possible that fungus-treated DCs increased the expression of these HIV-1 restriction factors and therefore become more resistant to HIV-1 infection.

DCs activate resting T cells and can provide more permissive targets for HIV-1 infection. We found that the stimulation of *P. marneffe* significantly accelerated DC-induced activation of resting CD4^+^ T cells, which indicates the pivotal importance of DC-driven T-cell activation for the high level of viremia and exacerbation of T-cell depletion in the late stage of HIV-1 infection. It would be interesting to confirm these *in vitro* observations in HIV-1-infected individuals.

Our findings revealed that DC function and its interaction with HIV-1 have been modulated by opportunistic pathogens for viral dissemination. Enhanced HIV-1 spread by DCs can target activated CD4^+^ T cells, which could further accelerate T-cell depletion and immunosuppression, leading to the lack of control of both viral and fungal pathogens. Our results highlight the importance of studying DC-HIV-1 interactions for understanding viral pathogenesis, and might provide a new insight into the interventions against HIV-1 infection and spread.

## Materials and Methods

### Ethics statement and fungi isolation and identification

This study was reviewed and approved by the Medical Ethics Review Committee of Yunnan Province, Kunming, China. Written informed consent was provided by study participants and/or their legal guardians. Fungi were isolated from skin lesions or tongue of AIDS patients and cultured on Sabouraud agar plates. Fungus species were identified, sub-cultured for amplification, then harvested and killed by boiling for 1 hr. Fungal cell counts were determined under a light microscope and diluted at 1×10^8^/ml in PBS. MDDCs were stimulated with fungi for 48 hrs at a 1∶10 ratio of cells.

### Cell culture

Human peripheral blood mononuclear cells (PBMCs) from healthy donors were provided by the Blood Center of Shanghai, Shanghai, China. CD14^+^ monocytes and resting CD4^+^ T cells were purified from PBMCs using magnetic beads (BD Biosciences) as described before [27]. CD14^+^ monocytes were cultured with granulocyte-macrophage colony-stimulating factor and interleukin (IL)-4 for 6 days to generate the immature DCs. Resting CD4^+^ T cells were activated with 5 µg/ml of phytohemagglutinin-P (PHA-P) (Sigma-Aldrich) for 48 h in the presence of 20 IU/ml of recombinant IL-2 (R&D). The human embryonic kidney cell lines HEK293T and the CD4^+^ T-cell line Hut/CCR5 (kind gifts from Dr. Vineet KewalRamani, National Cancer Institute, USA) have been described previously[27], [31], [32].

### HIV-1 or virus-like particle stocks

Pseudotyped single-cycle HIV-1 stocks were generated by using calcium phosphate-mediated co-transfection of HEK293T cells with the plasmid pLAI-Δ-env-Luc and expression plasmids of either JRFL (R5-tropic) or NL4-3 (X4-tropic) envelope glycoproteins as described previously [27], [31], [32]. Virus like particles (VLPs), HIV-1-Gag-GFP/JRFL, were generated by cotransfection of HEK293T cells with a plasmid encoding HIV-Gag-GFP and with an expression plasmid of JRFL (kind gifts from Dr. Vineet KewalRamani, National Cancer Institute, USA) [49]. Harvested supernatants of transfected cells that contained HIV-1 particles were filtered and titrated with p24^gag^ capture ELISA.

### Flow cytometry

Cells were stained with specific monoclonal antibodies (McAbs) or isotype-matched IgG controls. McAbs against the following human molecules were used for staining (clone numbers and resources are given in parentheses), Phycoerythrin (PE)-conjugated CD3 (UCHT1; eBioscience), PerCP-cy5.5-CD3 (OKT3, eBioscience), PE-CD11c (3.9; eBioscience), APC-Alexa Fluor750-CD11c (B-ly6; BD Pharmingen), PE-ICAM-1(CD54) (HA58;eBioscience), PE-CD69 (FN50; eBioscience), PE-CD83 (HB15e;eBioscience), PE-CD86 (IT2.2; eBioscience), PE-DC-SIGN (eB-h209; eBioscience), and APC-cy7-HLA-DR (LN3; eBioscience). Stained cells were detected with an LSRII flow cytometer (BD Pharmingen) and analyzed with FlowJo 7.6.1 software.

### HIV infection and transmission assays

The luciferase reporter system was adopted for assay of HIV-1 infection and transmission as previously described [27], [31], [32]. In brief, MDDCs were pulsed with 5 ng p24^gag^ amounts of pseudotyped HIV-luc/JRFL or HIV-luc/HXB2 for 2 h, and cells were washed for culture or for co-culture with CD4^+^ T cells. Hut/CCR5 or PHA-P-activated primary CD4^+^ T cells were used as targets. Cells were harvested after 3 days post-infection, and the cell lysates were measured for luciferase activity with a commercially available kit (Promega).

DC-mediated HIV-1 transmission also was detected by flow cytometry. HIV-1 VLP (HIV-Gag-GFP/JRFL) was used, and after 1-h co-culture of virus-loaded MDDCs with Hut/CCR5 cells, the T cells were distinguished based on CD11c^-^ staining from the mixed cell culture, and the numbers of Gag-GFP associated with T cells were measured.

### HIV-1 binding and internalization assay

HIV-1 binding and internalization were quantified by flow cytometry using the VLPs (HIV-Gag-GFP/JRFL). MDDCs were incubated with 40 ng p24^gag^ amounts of viruses for 2 h at 37°C and washed. The numbers of Gag-GFP associated with MDDCs were quantified by flow cytometry, and mean fluorescence intensity (MFI) was calculated. When indicated, anti- human DC-SIGN specific McAbs (10 µg/ml) (120507; Abcam) were used for pre-incubation with MDDCs before viral pulsing, or 5 min treatment with 0.25% trypsin (without EDTA) was used after viral-loading to remove surface-bound HIV-1.

### T-cells activation assay and viral infection

Various fungus-stimulated MDDCs or heat-killed fungi species were used to coculture with or treat allergenic resting CD4^+^ T cells for 48 h at the same ratio of cells. The T cells were gated based on CD3-positive populations, and the activation was monitored by detecting the transient expression of CD69 by flow cytometry. For viral infection, the activated T cells from DC-T cell co-cultures were purified by magnetic beads and then challenged with 5 ng p24^gag^ amounts of HIV-1/NL-43 for 2 h. After washing, the cells were further cultured for 5 days, and HIV-1 infection was detected with luciferase activity assay as mentioned above. Transwell culture plates with a membrane size of 0.4 µm were used to separate MDDCs and T cells.

### Confocal microscopy and image analysis

HIV-1 intracellular trafficking and the formation of virological synapses were observed by confocal microscopy. MDDCs were pulsed with HIV-1 VLPs, or virus-loaded MDDCs were co-cultured with CD4^+^ T cells for 30 min. Cells were seeded on the poly-L-lysine coated microscope slides and fixed with 4% paraformaldehyde (Sigma-Aldrich) for 1 h at 4°C. Cells were immunostained with anti-CD81 McAbs (M38; Abcam), anti-DC-SIGN McAbs (120507; R&D system), or anti-βactin (AC-15, Sigma), followed by staining with Alexa-Fluor 555-labeled goat anti-mouse IgG (Invitrogen). Nuclei were stained indicated with DAPI. Slides were mounted with Fluorescent Mounting Medium (Dako) and observed using a laser scanning confocal microscope (Leica SP5).

### Statistical analysis

Statistical analysis was performed using paired *t* test with the SigmaStat program.
